# 

**Published:** 1913-05-10

**Authors:** 


### Prussic Acid Poisoning.

What appears to be a "record" case of recovery from prussic acid poisoning is reported in the *Journal of the Royal Army Medical Corps* by Major Bourke. A private soldier procured from a chemist two drachms of the dilute hydrocyanic acid of the B.P., and swallowed the whole of it, except two or three drops. He fell unconscious on the floor of the barrack-room immediately. Fortunately two non-commissioned officers of the Army Medical Corps were in the next room, who commenced artificial respiration and telephoned for their officer. When the latter arrived he did not think recovery of the patient was possible: the pulse was not perceptible, the face was livid, respiration convulsive and finally absent. Artificial respiration, emesis, amyl nitrite inhalations, and atropine hypodermically eventually pulled the patient through after many convulsions during an anxious two hours. The chief interest in the case lies in the dose of poison which was self-administered. Taylor's "Medical Jurisprudence" gives the largest dose after which recovery has been recorded as one drachm of the dilute acid. Fifty drops are mentioned as a dose which ordinarily destroys life in an adult; and two drachms, it is opined, will destroy life in two to ten minutes. The pure anhydrous acid, of course, is fatal in doses of one drop or so. It seems just possible that in Major Bourke's case the dilute acid may not have been quite up to standard strength.

### Buildings Contemplated.

Blackrod.—Preliminary plans have been adopted by the Urban District Council for the erection of a hospital for smallpox and phthisis cases. The estimated cost is £4,000.

Durham.—The question of a site for a new workhouse hospital is under consideration by the Guardians.

Chester.—The Local Government Board has held an inquiry into an application of the Corporation for sanction to a loan of £39,894 in connection with the Upton Lunatic Asylum. The plans are by Mr. H. Beswick, the county architect.

Conway.—An inquiry has been held by the Local Government Board into applications by the Conway and Penmaenmawr Joint Hospital Board for permission to borrow £2,508 in connection with the infectious diseases hospital at Croesynyd, and £600 for the purchase of certain hospital buildings at Lysfaen. The plans have been prepared by Messrs. T. B. Farrington and Sons, Trinity Square, Llandudno.

Eastbourne.—It is proposed by the Corporation to build a new hospital for consumption at an estimated cost of £4,500.

Greenwich.—The Borough Council have decided to expend the sum of £550 on purchasing the freehold of Manor House, Park Place, Greenwich, for a tuberculosis dispensary.

Hemel Hempstead.—Instructions have been issued by the Hemel Hempstead Joint Hospitals Board to Messrs. Snell and Spoor to prepare plans, for submission to the Local Government Board, of a new isolation hospital. It is estimated that the building will cost £5,235.

Hitchin.—The Rural District Council have agreed to apply to the Local Government Board for sanction to borrow £1,050 for a site for an isolation hospital.

Rhymney.—A deputation has been appointed by the Urban District Council to confer with the County Council in regard to the proposed joint isolation hospital.

### A Curious Salvarsan Case.

A very strange case, and difficult of explanation, is reported in the *Journal of the American Medical Association.* Eight years ago a man consulted his doctor for indubitable syphilis of three weeks' duration. He was treated steadily for three years by mercury in the usual way of those days. Five years after being first seen he consulted his doctor again because he had heard of the Wassermann reaction and wished to have himself tested by it. A negative report was the result of the test. Nine months later he wished to have the reaction again tried, and once more it was negative. A year later he showed signs of commencing tabes dorsalis, and that diagnosis seems to be incontrovertible. He was then given 0.6 gm. of salvarsan intravenously. Four days later a minute erosion developed on the glands, which was at first regarded as herpetic in nature. A week later the sore looked like a chancre, and the inguinal glands were enlarged; spirochætes could not be found in the sore. Four days later the spirochætes were demonstrated however; and a syphilitic skin eruption developed. The tabetic symptoms remained unchanged. The Wassermann reaction was found to be positive. It is certainly most extraordinary that a tabetic patient, apparently cured of his original syphilis, should develop a second attack just after a full dose of salvarsan, and that, in spite of the latter, the secondary manifestations should follow with such rapidity upon the primary sore.

### Obituary.

Mr. Matthew Algernon Adams, F.R.C.S., the inventor of the horamagraph, has died, aged seventy-seven. For many years medical officer of health for Maidstone and public analyst for the town and county, he was consulting surgeon to the Maidstone Ophthalmic Hospital. His previous institutional appointments included that of senior resident surgeon at Leeds Public Dispensary.

Dr. John Charles Thorowgood has died at the age of eighty-one. Educated at University College, where he won the gold medal for chemistry and medicine, he qualified L.R.C.S., L.S.A., and eventually graduated M.B. and M.D. of London. He became a Fellow of the Royal College of Physicians and consulting physician to the West London Hospital, the City of London Chest Hospital, and the Royal National Hospital for Consumption. In 1879 his Lettsomian lectures on bronchial asthma were published, and this subject, together with the climatic treatment of phthisis, was dealt with in several publications by him.

### Gifts and Bequests.

Mrs. Stretton has sent a contribution of £50 to the Leicester Children's Hospital.

Dr. Barnardo's Homes have received 30 guineas from the Merchant Taylors' Company.

The Royal Dental Hospital, Leicester Square, has received £100 from Mr. John Lewis, executor of the will of Miss Ames.

The Chelsea Hospital for Women has received £31 10s. from the Merchant Taylors' Company towards its rebuilding fund.

A legacy of £230 is reported from a working man, who was a patient at the Willesden Cottage Hospital, to that institution, in its annual report.

The Chelsea Hospital for Women has received £5 5s. from the Companies of Salters and Armourers and Braziers; and a bequest of £25 from the late Miss E. C. Laurence, for some years matron of the hospital.

Miss Margaret Jane Ashley, of Rowan House, Dorchester, a niece of Lord Shaftesbury, the philanthropist, has left £1,000 to the Dorset County Hospital and £500 to the Weymouth Eye Infirmary.

The Committee of Management of the Great Northern Central Hospital acknowledge £100 from Miss M. W. Mitchell towards the fund for rebuilding the Nurses' Home.

Among the latest contributions to King Edward's Hospital Fund are the following annual subscriptions: Messrs. Speyer Brothers, £250; Mr. A. M. Singer, £100; Lord Wemyss and March, £50; and donations: "A.," £1,000; Messrs. Crosse and Blackwell, Limited, £52 10s.

Property of the gross value of £947,633 has been left by Mr. Edward Webb, Stourbridge, seedsman and hop merchant, who left £5,000 to the Corbett Hospital, Amblecote, Staffs, as to £3,000 for or towards a wing to be called the Margaret Jane Webb Wing in memory of his wife, and £2,000 for its furnishing and equipment.

Miss Julia Susanna Rogers, of Hampstead, has left £1,000 each to the Blind Asylum, Hampstead, and Miss Weston's Home of Rest, Portsmouth; £500 to the Devonshire Hospital at Buxton; and £200 to the Seaside Branch of the Metropolitan Convalescent Institution, Bexhill.

Mrs. Mary Catherine Elizabeth Rogers, of Bath, has left £1,200 to the Mildmay Memorial Hospital at Bethnal Green, and £200 to the Mildmay Memorial Cottage Hospital, Stoke Newington, for the endowment of "James Watson" beds; £500 to Gilbert Bannatyne for such convalescent home as he shall choose; and £1,000 to the trustees of her will for distribution among charitable objects in Bath.

Miss Kate Brockmer Brockmer, of Purton, Wilts, has bequeathed £500 to St. Luke's Home for the Dying, Bayswater, and the residue of her estate, subject to the payment of certain legacies, she left in equal shares to the Church Missionary Society, the Royal Hospital for Incurables, Putney, and Dr. Barnardo's Homes. The amount available for charitable purposes appears to be about £9,000.

### £120,000 FOR FRENCH HOSPITALS.

Madame Debrousse, whose death was announced recently, has made a universal legacy in favour of the Assistance Publique. She has expressed the wish that a sum of 1,500,000 francs (£60,000) shall be consecrated to the enlargement of the Hôpital Trousseau, and that another similar amount shall be employed in the enlargement of the Hospice Debrousse.

### A UNIQUE RAILWAY ADVERTISEMENT.

A railway advertisement of unique character has recently been produced by the Great Eastern Railway Company. It takes the form of a parody, in prose, verse, and picture, on Lewis Carroll's immortal heroine, "Alice in Wonderland," under the suggestive title of "Alice in Holidayland." Great pains and considerable skill have been exercised in the production of the booklet. On the cover appear the familiar figures such as the Walrus and Carpenter, Alice, the Mock Turtle, and the Gryphon, and inside are twelve excellent coloured plates illustrating the parody. The story cleverly introduces the advantages of holiday resorts, more especially for children, of such places as Scarborough, Whitby, Bridlington, Withernsea, and Filey, all of which can most conveniently be reached by the Company's line. A copy of the booklet can be obtained upon application to the passenger manager's office of the Great Eastern Railway at York, or from station bookstalls.

### Appointments.

The Metropolitan Asylums Board has appointed Dr. William Mair as research pathologist at a salary of £500 per annum. The appointment is for one year in the first instance.

Dr. G. H. Lancashire, M.D., M.R.C.S., L.R.C.P., has been appointed lecturer on the diseases of the skin at Manchester University. Dr. Lancashire is honorary physician to the Manchester and Salford Hospital for Diseases of the Skin.

The other tuberculosis officers for the county of Middlesex under the new scheme, besides Dr. E. E. Norton, whose appointment we announced last week, are Dr. F. R. B. Atkinson, Dr. Harry Coghill, Dr. J. R. B. Dobson, and Dr. D. G. M. Munro.

Dr. C. P. Lapage, M.D., M.R.C.P., has been appointed lecturer at Manchester University on the diseases of children. Dr. Lapage holds the position of visiting physician to the Manchester Children's Hospital. He has published a work on feeble-mindedness in children and articles on children's diseases in "The Encyclopædia of Medicine and Surgery."

The following appointments have been made at the Manchester Royal Infirmary: Mr. James Walker, M.B., Ch.B. (Vict.), as assistant director of the clinical laboratory; Mr. D. L. Sewell, M.B., B.S. (Lond.), M.R.C.S., L.R.C.P., assistant surgical officer to the aural department; Mr. J. C. Jefferson, M.B., B.S. (Lond.), M.R.C.S., L.R.C.P., as medical officer at the central branch. Re-appointments: Mr. G. M. Benton, M.B., Ch.B. (Vict.), as sixth anæsthetist; Mr. George Ashton, M.D., as medical officer for home patients; Mr. C. H. Melland, M.R.C.P., Mr. F. E. Tylecote, M.R.C.P., and Mr. E. B. Leech, M.R.C.P., as assistant medical officers; and Mr. W. R. Douglas, F.R.C.S., as assistant surgical officer. Dr. A. T. Wilkinson has been appointed honorary consulting physician to the institution, and Dr. E. N. Cunliffe, assistant hon. physician, has been appointed hon. physician to fill the vacancy.

### Personalia.

A pleasing gift of £52 10s. to the Leicester Children's Hospital has been made by Mrs. Bowmar, in gratitude for the skill which Dr. Pratt has shown to her and of his long service as hon. physician to the Leicester Royal Infirmary.

It is now thirty-four years since Dr. Purey-Cust was appointed Dean of York, and it has been decided to commemorate his public services. A sum of £2,128 has been subscribed, and at the Dean's request will be devoted to the erection of a nursing institute, to cost £5,000, to be christened after him. The Dean of York is, of course, chairman of the York County Hospital.

Reference was made in The Hospital of April 19 to the apparent difficulty which was being experienced in finding sufficient members willing to serve upon the London Mental Hospitals Committee, which under standing orders should consist of thirty to thirty-five members. It was then stated that of the twenty-nine members who had been appointed, four had resigned, and since that date Mr. D. Davies and Mr. G. A. Hardy have tendered their resignations. Mr. Hardy, however, has been persuaded to withdraw his resignation, but there is no indication at present of how the other vacancies are to be filled.

Lord St. Aldwyn has been elected to act as president for one year of the Cheltenham General Hospital.

The Honourable Arthur Lyulph Stanley has joined the General Committee of University College Hospital.

Prince Arthur of Connaught has consented to become president of the West London Hospital, in succession to the late Duke of Abercorn.

The salary of Dr. Rotheram, the medical superintendent at Darenth Industrial Colony, has been increased from £800 to £900 a year.

The Metropolitan Asylums Board has increased the salary of Dr. Pugh, medical superintendent at Queen Mary's Hospital, from £600 to £700 per annum.

Miss Frances Margaret Harper, M.B., B.S., has been awarded the diploma in tropical medicine and hygiene granted by the Royal Colleges of Physicians and Surgeons of London. She is the first woman to receive this distinction. She holds the appointment of outdoor house surgeon at the West-End Branch of the Glasgow Maternity Hospital.

The board of the General Hospital, Leeds, have recorded in a special resolution their deep regret at Mr. Littlewood's resignation, and their appreciation of the great services which he has rendered to the patients and to surgery. For twenty-three years he has served as assistant surgeon and surgeon, and the weekly board recommend his election as consulting surgeon to the institution at the next general meeting of governors.

### Books Received.

C. W. Daniel, Ltd.: "Unfired Food in Practice," by Stanley Gibbon.

The Clarendon Press: "First Principles of Hygiene," by W. D. Sturrock.

T. C. and E. C. Jack: "The Parents' Book."

Chapman and Hall: "The Night Nurse," by the Author of "The Surgeon's Log."

T. Nelson and Sons: "The Book of Diet," by Dr. Chalmers Watson.

George Pulman and Sons: "Sclero-Corneal Trephining in the Operative Treatment of Glaucoma," by Robert Elliot, M.D.; "Eye Strain in Everyday Practice," by Sydney Stephenson.

J. and A. Churchill and Sons: "Bacteria and Protozoa," by Herbert Fox, M.D.

J. Wright and Sons: "The Medical Annual, 1913."

### SUMMER SEA TRIPS BY ORIENT LINE STEAMERS.

THE Orient Line announce a reduction in fares during the summer season on their 15-day sea trips to the South of Spain and the Riviera. The saloon fare for the 15-day round trip to Toulon is reduced to £15 and the second saloon fare to £10 10s. An illustrated programme of these trips can be obtained from the Orient Line Offices.

